# Rewriting the prognosis of multiple sclerosis in Brazil: a 25-year perspective on evolving diagnostic criteria

**DOI:** 10.1055/s-0045-1809937

**Published:** 2025-07-02

**Authors:** Dagoberto Callegaro, Guilherme Diogo Silva

**Affiliations:** 1 Universidade de São Paulo, Hospital das Clínicas, Faculdade de Medicina, Departamento de Neurologia, São Paulo SP, Brazil.

In this issue of *Arquivos de Neuropsiquiatria* , Menezes et al. 1 investigate whether the progressive adoption of more sensitive diagnostic criteria – from Poser through successive McDonald revisions – may be associated with less disability accumulation in patients with relapsing-remitting multiple sclerosis. To investigate this, the authors conducted a 25-year retrospective cohort study of 491 patients diagnosed between 1994 and 2019 in Sao Paulo, Brazil. This question is important because multiple sclerosis is the most common relapsing demyelinating disease of the central nervous system, 2 and its prognosis may have changed over the past 25 years.

A key finding of Menezes et al. was that patients diagnosed in Epoch 3 (2011–2019) - McDonald 2010 and 2017's criteria - had a 63% lower risk of reaching EDSS 6.0 than those in Epoch 1 (1994–2001) - Posner's criteria. One explanation is that the newer diagnostic criteria reduced the median time to first disease-modifying therapy from three to two years and dramatically increased the proportion of patients treated before their second relapse (from 2.7% to 24.4%). By enabling diagnosis at the very first demyelinating event – such as optic neuritis, for which multiple sclerosis is a leading cause 3 –these more sensitive criteria ensure patients start treatment sooner, further minimizing early disability accrual.

A second contributing factor may be the introduction of high-efficacy therapies. A meta-analysis of 3,467 participants showed that early use of high-efficacy agents was associated with a 30% reduction in the risk of EDSS worsening at five years compared with escalating strategy. 4 5 Likewise, the Brazilian MS Cohort (BRANDO) found that regions with higher average EDSS scores had lower rates of high-efficacy prescriptions – such as natalizumab – underscoring the impact of access to high-efficacy treatments. 6 While more sensitive diagnostic criteria can raise concerns about misdiagnosis and overtreatment, real-world data from MS referral centers show that misdiagnosis with the 2017 McDonald criteria remains low. 7 Furthermore, these centers follow standardized care protocols – endorsed by the Brazilian Academy of Neurology and the Brazilian Committee for Treatment and Research in Multiple Sclerosis (BCTRIMS) – to proactively monitor and minimize treatment-related adverse events. 8 9

Another notable finding of Menezes et al.'s study was a 53% lower hazard of conversion to SPMS in the most recent epoch compared to the Poser era. This is especially significant given the limited efficacy of both on-label and off-label disease-modifying therapies in preventing disability accrual in progressive MS. 10

Ultimately, the dramatic decline in disability progression and SPMS conversion over time likely reflects three game-changing advances in Brazil: the adoption of more sensitive diagnostic criteria enabling earlier recognition, the slashing of delays to treatment initiation, and the vastly expanded access to high-efficacy therapies. Together, these shifts have fundamentally reshaped the MS prognosis – demonstrating that when diagnosis is early and potent DMTs are within reach, the trajectory of this disease can indeed be rewritten.
